# Retraction: Isolated Superior Mesenteric Artery Dissection After Methamphetamine Use: A Rare Adverse Effect

**DOI:** 10.7759/cureus.r96

**Published:** 2024-01-25

**Authors:** Rayan A Alzahrani, Omar M Alzahrani, Sarah H Alherz, Abdulrahman A Alzahrani, Omran M Alzahrani, Mohammed A Alqarni, Khalid Abdulrahman A Alzahrani, Khalid Abdulkarim H Alzahrani, Suhayb A Al Zahrani, Abdulaziz A Alzahrani, Hamad A Alghamdi, Malak Alshammari

**Affiliations:** 1 College of Medicine, Al-Baha University, Al-Baha, SAU; 2 College of Medicine, Imam Abdulrahman Bin Faisal University, Dammam, SAU

The Editors-in-Chief have retracted this article. Concerns were raised regarding the identity of the authors on this article. Specifically, Faisal Alhaway and Malak Shammari have stated that they were added to this article without their knowledge or approval. The identity of the other authors could also not be verified. As the appropriate authorship of this work cannot be established, the Editors-in-Chief no longer have confidence in the results and conclusions of this article.

